# Supplementary material for: Differential Cytokine Responses and the Clinical Severity of Adult and Pediatric Nephropathia Epidemica
Source: Int J Mol Sci. 2023 Apr 10;24(8):7016. doi: 10.3390/ijms24087016 (PMC10139191; doi:10.3390/ijms24087016)
Supplement: Supplementary file 1 [file ijms-24-07016-s001.zip › ijms-2158045-supplementary.pdf]

Table S1. List of the obtained PUUV strains and GenBank accession No of S segment sequences.

Strain	Short Name	Accession No
PUUV/Kazan/human_RT465/2019/segment S	Hu465	OQ067390
PUUV/Kazan/human_RT585/2019/segment S	HU585	OQ067391
PUUV/Kazan/human_RT600/2019/segment S	Hu600	OQ067392
PUUV/Kazan/human_RT626/2019/segment S	HU626	OQ067393
PUUV/Kazan/human_RT627/2019/segment S	HU627	OQ067394

Table S2. List of the PUUV sequences used for phylogenetic analysis as references.

Strain sequence	Short Name	GenBank Accession No
PUUV/Vysokaya Gora/MG_066/2015/segment S		MZ913276
PUUV/Observatory/MG_118/2015/segment S		MW587801
PUUV/Kazan/MG_845/2017/segment S		MW587804
PUUV/Pestretsy/MG_273/2015/segment S		MW504248
PUUV/Lenino-Kokushkino/MG_1140/2017/segment S		MW504252
PUUV/Staraya Pristan/MG_1675/2019/segment S		MW504256
PUUV/Laishevo/MG_809/2017/segment S		MW504247
PUUV/Sotyi/MG_952/2017/segment S		MZ441152
PUUV/Mamadysh/MG_980/2017/segment S		MW504250
PUUV/Verkhniaya Oshma/MG_1215/2018/segment S		MW504253
PUUV/Teteevo/MG_1041/2017/segment S		MZ441151
PUUV/Naberezhnye Chelny/MG_260/2015/segment S		MW504226
PUUV/Nizhnekamsk/MG_134/2015/segment S		MW504222
PUUV/Almetievsk/MG_1182/2018/segment S		MW504212
PUUV/Tatarskoe Utiashkino/MG_1419/2019/segment S		MW504213
PUUV/Kurkul/MG_1459/2019/segment S		MW504214
PUUV/Oktiabrino/MG_1469/2019/segment S		MW504215
PUUV/Chistopolskie Vyselki/MG_2030/2020/segment S		MW504224
PUUV/Galaktionovo/MG_2038/2020/segment S		MW504245
PUUV/Starye Salmany/MG_1589/2019/segment S		MW504240
PUUV/Verkhnee Almurzino/MG_1613/2019/segment S		MW504218
PUUV/Lesnye Morkvashi/MG_794/2017/segment S		MW587803
Puu/Kazan		Z84204
DTK/Ufa-97		AB297665
Samara_49/CG/2005		AB433843
Sotkamo 2009		HE801633
Tula virus		EU439951
PUUV/Kazan/human_RT458/2019/segment S	Hu458	MW587805
PUUV/Kazan/human_RT461/2019/segment S	Hu461	MW587793
PUUV/Kazan/human_RT464/2019/segment S	Hu464	MZ913278
PUUV/Kazan/human_RT466/2019/segment S	Hu466	MZ913279
PUUV/Kazan/human_RT471/2019/segment S	Hu471	OP459274
PUUV/Kazan/human_RT475/2019/segment S	Hu475	MZ913280
PUUV/Kazan/human_RT487/2019/segment S	Hu487	OP459275
PUUV/Kazan/human_RT488/2019/segment S	Hu488	MW587794
PUUV/Kazan/human_RT493/2019/segment S	Hu493	MW587795
PUUV/Kazan/human_RT497/2019/segment S	Hu497	MW587796
PUUV/Kazan/human_RT500/2019/segment S	Hu500	MW587808

PUUV/Kazan/human_RT505/2019/segment S	Hu505	MW587797
PUUV/Kazan/human_RT518/2019/segment S	Hu518	MW587798
PUUV/Kazan/human_RT520/2019/segment S	Hu520	MW587799
PUUV/Kazan/human_RT523/2019/segment S	Hu523	MZ913281
PUUV/Kazan/human_RT526/2019/segment S	Hu526	MW587809
PUUV/Kazan/human_RT542/2019/segment S	Hu542	OP459295
PUUV/Kazan/human_RT545/2019/segment S	Hu545	MW587810
PUUV/Kazan/human_RT546/2019/segment S	Hu546	MW587800
PUUV/Kazan/human_RT549/2019/segment S	Hu549	OP459278
PUUV/Kazan/human_RT563/2019/segment S	Hu563	MZ913284
PUUV/Kazan/human_RT566/2019/segment S	Hu566	OP459279
PUUV/Kazan/human_RT574/2019/segment S	Hu574	MW587800
PUUV/Kazan/human_RT587/2019/segment S	Hu587	OP459283
PUUV/Kazan/human_RT598/2019/segment S	Hu598	OP459285
PUUV/Kazan/human_RT599/2019/segment S	Hu599	OP459286
PUUV/Kazan/human_RT602/2019/segment S	Hu602	OP459287
PUUV/Kazan/human_RT603/2019/segment S	Hu603	OP459288
PUUV/Kazan/human_RT604/2019/segment S	Hu604	MZ913285
PUUV/Kazan/human_RT608/2019/segment S	Hu608	OP459289
PUUV/Kazan/human_RT611/2019/segment S	HU611	OP459290
PUUV/Kazan/human_RT614/2019/segment S	Hu614	OP459291
PUUV/Kazan/human_RT624/2019/segment S	Hu624	OP459292
PUUV/Kazan/human_RT638/2019/segment S	Hu638	MZ913286
PUUV/Kazan/human_RT639/2019/segment S	HU639	OP459294

Table S3. Analysis of signs and symptoms in adult and children NE.

**Feature**		**Adult NE**	**Children NE**	***p* Value**
Severity		2.05 ± 0.38	2.09 ± 0.30	0,77
Fever, days		6.26 ± 2.45	6.64 ± 3.50	0.83
2nd fever, days		1.01 ± 2.21	0.00 ± 0.00	**0.04** *
Headache, days		4.52 ± 2.91	1.27 ± 2.57	**0.001** *
Cough, days		0.73 ± 2.34	0.00 ± 0.00	0.17
Nausea, days		1.19 ± 1.81	0.55 ± 0.52	0.91
Vomit, days		0.53 ± 1.08	0.45 ± 0.52	0.48
Abdominal pain, days		1.15 ± 1.87	0.64 ± 0.92	0.86
Diarrhea, days		0.78 ± 1.51	0.09 ± 0.30	0.08
Lumbar pain, days		4.47 ± 3.43	3.00 ± 5.33	**0.03** *
Oliguria, days		2.22 ± 1.92	1.00 ± 1.41	**0.03** *
Anuria, days		0.06 ± 0.32	0.00 ± 0.00	0.51
Urea, mMdL	Febrile	8.39 ± 6.03	5.18 ± 1.99	0.07
	Oliguric	10.13 ± 7.50	10.29 ± 9.95	0.35
	Polyuric	6.44 ± 8.87	5.28 ± 1.11	0.98
sCr, mM/L	Febrile	148.85 ± 112.36	70.55 ± 17.33	**<0.0001** *
	Oliguric	172.64 ± 141.60	144.64 ± 118.82	0.14
	Polyuric	103.60 ± 31.93	75.18 ± 28.85	**0.01** *
K, mEq/L	Febrile	4.04 ± 0.43	4.06 ± 0.28	0.96
	Oliguric	5.43 ± 11.91	4.40 ± 0.50	0.99
Thrombocytes, cells/μL	Febrile	90.71 ± 56.30	91.73 ± 51.39	0.96
	Oliguric	143.37 ± 82.75	150.45 ± 69.70	0.56
	Polyuric	256.71 ± 83.75	266.55 ± 68.31	0.65

ALT, Ed/L	Febrile	50.90 ± 47.00	38.00 ± 29.62	0.26
	Oliguric	58.53 ± 61.80	37.82 ± 25.02	0.15
	Polyuric	61.17 ± 66.54	39.82 ± 30.96	0.12
AST, Ed/L	Febrile	54.96 ± 40.65	67.27 ± 31.93	0.07
	Oliguric	52.90 ± 39.69	54.00 ± 19.43	0.20
	Polyuric	45.90 ± 28.01	43.64 ± 15.95	0.72
Leukocytes, cells/μL	Febrile	9.56 ± 5.47	7.80 ± 4.19	0.19
	Oliguric	10.54 ± 4.30	10.14 ± 3.56	0.80
	Febrile	8.14 ± 2.20	7.31 ± 1.44	0.27
Hemoglobin, g/L	Oliguric	146.17 ± 22.92	142.27 ± 17.92	0.39
	Polyuric	137.22 ± 20.03	132.73 ± 11.41	0.33
	Febrile	139.95 ± 16.00	135.64 ± 10.48	0.27

* *p* adj. < 0.05 when compared with controls.

Table S4. Analysis of serum cytokines level in adult NE.

Marker		Control, pg/mL	NE (Febrile Phase), pg/mL	NE (Oliguric Phase), pg/mL	NE (Polyuric Phase), pg/mL
Chemokines	CCL27	13.79 ± 4.46	**66.65 ± 21.04 ***	**70.63 ± 37.86 ***	**59.98 ± 29.38 ***
	CCL11	11.44 ± 10.98	9.42 ± 8.96	8.40 ± 6.59	7.99 ± 7.61
	G-CSF	34.68 ± 26.35	243.79 ± 321.75	182.13 ± 287.51	165.49 ± 270.70
	GM-CSF	31.87 ± 5.21	**64.89 ± 12.29 ***	**63.43 ± 9.77 ***	**62.00 ± 8.90 ***
	CXCL1	186.23 ± 41.24	**388.57 ± 22.60 ***	**387.78 ± 33.63 ***	**386.86 ± 43.58 ***
	HGF	213.14 ± 106.47	187.24 ± 104.84	**147.06 ± 68.15 ***	177.47 ± 92.25
	IFN-α2	22.35 ± 5.71	**69.17 ± 14.52 ***	**71.57 ± 11.10 ***	**71.04 ± 10.30 ***
	IFN-γ	63.83 ± 7.67	69.20 ± 13.63	65.27 ± 13.69	66.80 ± 16.71
	CXCL10	123.98 ± 18.71	148.70 ± 34.24	**152.34 ± 39.09 ***	**148.46 ± 38.57 ***
	LIF	183.61 ± 35.36	**239.10 ± 72.19 ***	**316.15 ± 367.80 ***	**280.32 ± 52.77 ***
	M-CSF	42.95 ± 6.92	**73.88 ± 9.12 ***	**74.32 ± 12.28 ***	**74.45 ± 11.60 ***
	CCL2	171.49 ± 40.24	**366.42 ± 65.95 ***	**349.60 ± 54.42 ***	**345.02 ± 67.96 ***
	CCL7	56.20 ± 12.71	50.25 ± 9.45	50.49 ± 11.60	49.37 ± 10.67
	MIF	134.70 ± 60.44	127.78 ± 47.33	125.02 ± 51.94	121.25 ± 49.03
	CXCL9	61.10 ± 26.09	**160.43 ± 36.17 ***	**155.78 ± 43.57 ***	**157.22 ± 31.18 ***
	CCL3	19.10 ± 24.94	**51.80 ± 46.02 ***	**43.77 ± 46.09 ***	**42.56 ± 42.61 ***
	CCL4	13.87 ± 4.02	**19.70 ± 7.64 ***	**20.29 ± 9.10 ***	**17.30 ± 6.32 ***
	PDGF-BB	151.66 ± 62.01	179.07 ± 59.33	171.98 ± 43.17	170.23 ± 51.45
	CCL5	183.05 ± 206.19	**372.46 ± 272.22 ***	**373.55 ± 262.26 ***	**308.84 ± 226.01 ***
	SCF	33.40 ± 44.72	25.19 ± 34.57	31.26 ± 44.53	23.08 ± 21.37
	SCGF-b	7569.40 ± 6079.21	12145.38 ± 11662.96	12186.00 ± 9873.16	11855.44 ± 9934.26
	SDF-1a	31.13 ± 10.24	43.02 ± 28.16	46.63 ± 28.05	38.14 ± 28.84
	TNF-a	34.51 ± 7.78	**78.59 ± 18.73 ***	**76.64 ± 16.11***	**82.27 ± 16.15 ***
	TNF-b	23.62 ± 39.13	37.16 ± 49.46	44.38 ± 54.02	30.05 ± 41.82
	TRAIL	26.92 ± 6.90	**44.53 ± 8.11 ***	**46.07 ± 8.60 ***	**46.66 ± 11.48 ***
	VEGF	204.85 ± 48.57	**428.98 ± 40.64 ***	**425.22 ± 58.89 ***	**442.73 ± 62.34 ***
	b-NGF	20.22 ± 5.11	20.00 ± 5.05	18.48 ± 5.26	18.38 ± 5.17
	bFGF	62.24 ± 11.80	63.60 ± 11.82	69.13 ± 51.61	67.73 ± 12.93
Interleukins	IL-1Ra	97.87 ± 92.23	123.77 ± 167.31	191.10 ± 204.95	155.03 ± 192.33
	IL-1α	84.76 ± 15.19	85.78 ± 14.40	83.73 ± 16.58	79.00 ± 16.57
	IL-1β	4.74 ± 8.06	**21.08 ± 3.04 ***	**24.37 ± 8.67 ***	**24.63 ± 13.32 ***
	IL-2	130.70 ± 13.09	**146.00 ± 12.84 ***	**144.76 ± 11.70 ***	**144.36 ± 10.73 ***
	IL-2Ra	93.19 ± 8.69	**118.53 ± 26.54 ***	**121.16 ± 15.98 ***	**117.22 ± 17.37 ***
	IL-3	11.06 ± 0.63	**13.41 ± 0.85 ***	**13.49 ± 0.93 ***	**17.22 ± 21.07 ***

	IL-4	8.93 ± 5.54	**11.62 ± 2.99 ***	**12.44 ± 3.29 ***	**14.32 ± 9.02 ***
	IL-5	208.17 ± 46.07	**409.50 ± 31.15 ***	**401.68 ± 38.22 ***	**404.29 ± 35.16 ***
	IL-6	28.76 ± 5.03	29.99 ± 5.60	29.05 ± 5.38	33.22 ± 33.15
	IL-7	135.56 ± 52.55	**173.68 ± 10.52 ***	**174.80 ± 8.71***	**175.14 ± 9.31 ***
	IL-8	22.90 ± 18.20	36.46 ± 5.62	36.43 ± 4.46	37.65 ± 4.04
	IL-9	7.78 ± 7.33	**16.23 ± 3.56 ***	**17.31 ± 5.80 ***	**15.62 ± 3.77 ***
	IL-10	30.77 ± 6.25	**64.91 ± 8.90 ***	**65.04 ± 8.37 ***	**65.17 ± 9.42 ***
	IL-12p40	420.69 ± 69.56	**655.23 ± 81.66 ***	**642.94 ± 89.91 ***	**645.02 ± 105.99 ***
	IL-12p70	296.14 ± 19.86	318.40 ± 46.88	320.07 ± 53.87	**349.76 ± 67.48 ***
	IL-13	28.59 ± 4.22	29.93 ± 6.57	30.68 ± 5.19	28.75 ± 4.79
	IL-15	973.38 ± 205.59	**1365.60 ± 63.18 ***	**1566.65 ± 1536.77 ***	**1401.17 ± 199.06 ***
	IL-16	73.49 ± 14.23	**108.52 ± 33.49 ***	**113.51 ± 34.13 ***	**108.98 ± 29.44 ***
	IL-17	138.92 ± 11.76	**210.64 ± 29.28 ***	**200.20 ± 27.54 ***	**195.01 ± 24.16 ***
	IL-18	65.27 ± 6.04	**76.06 ± 15.24 ***	**82.71 ± 16.05 ***	**82.27 ± 15.30 ***
Urine kidney damage mark-ers	Calbindin	41.12 ± 23.56	**27.24 ± 59.65 ***	**16.43 ± 26.88 ***	**17.70 ± 30.97 ***
	GST-p	1.37 ± 1.69	16.44 ± 49.13	8.62 ± 22.46	4.67 ± 7.28
	IL-18	1.64 ± 1.77	2.07 ± 5.47	8.17 ± 44.75	1.74 ± 5.14
	KIM-1	0.86 ± 0.89	0.65 ± 1.10	1.29 ± 4.01	1.60 ± 5.11
	MCP-2	1.49 ± 2.21	0.94 ± 1.65	1.18 ± 3.25	1.61 ± 4.13
	Clusterin	40.43 ± 70.92	**383.60 ± 823.43 ***	**505.09 ± 789.47 ***	**431.15 ± 805.62 ***

* p adj. < 0.05 when compared with Controls.

Table S5. Analysis of serum cytokines level in pediatric NE.

**Marker**		**Control, pg/mL**	**NE (Febrile Phase), pg/mL**	**NE (Oliguric Phase), pg/mL**	**NE (Polyuric Phase), pg/mL**
Chemokines	CCL27	76.29 ± 6.67	**187.91 ± 17.83 ***	**268.83 ± 191.06 ***	**218.82 ± 104.64 ***
	CCL11	7.45 ± 3.44	**23.19 ± 11.56 ***	**16.83 ± 4.40 ***	**19.61 ± 1.90 ***
	G-CSF	184.74 ± 23.82	195.19 ± 36.55	188.92 ± 36.31	202.73 ± 16.85
	GM-CSF	61.07 ± 11.36	62.41 ± 11.60	57.57 ± 9.71	58.09 ± 16.12
	CXCL1	404.05 ± 22.78	406.52 ± 24.70	394.21 ± 33.17	426.00 ± 48.05
	HGF	190.77 ± 67.23	159.55 ± 36.05	163.59 ± 36.70	187.15 ± 32.66
	IFN-α2	54.73 ± 7.84	53.22 ± 8.15	56.83 ± 8.23	57.36 ± 4.49
	IFN-γ	79.26 ± 10.53	73.52 ± 21.01	81.89 ± 9.97	73.24 ± 15.05
	CXCL10	60.94 ± 15.78	**202.15 ± 49.47 ***	**160.79 ± 39.28 ***	162.22 ± 6.45
	LIF	300.41 ± 17.30	292.94 ± 41.13	252.92 ± 67.16	269.78 ± 30.16
	M-CSF	59.30 ± 10.70	52.80 ± 2.57	68.05 ± 7.05	61.46 ± 14.93
	CCL2	25.74 ± 5.32	**45.30 ± 5.22 ***	**58.66 ± 25.00 ***	**49.20 ± 0.79 ***
	CCL7	41.51 ± 2.35	44.59 ± 3.42	39.35 ± 9.38	36.44 ± 10.53
	MIF	176.65 ± 39.97	**223.86 ± 28.00 ***	175.72 ± 35.22	194.99 ± 10.00
	CXCL9	38.27 ± 18.25	58.46 ± 20.09	65.25 ± 25.96	78.30 ± 24.48
	CCL3	7.91 ± 1.44	8.71 ± 1.16	7.83 ± 0.95	6.51 ± 0.48
	CCL4	11.67 ± 5.76	24.14 ± 10.50	**31.63 ± 10.66 ***	26.22 ± 6.21
	PDGF-BB	126.44 ± 65.79	194.54 ± 23.76	185.87 ± 38.77	141.85 ± 14.21
	CCL5	199.60 ± 194.63	157.34 ± 37.56	347.85 ± 228.94	160.19 ± 50.97
	SCF	42.11 ± 25.81	82.96 ± 50.34	79.47 ± 22.40	**137.52 ± 28.57 ***
	SCGF-b	2794.56 ± 1845.40	7457.16 ± 9822.63	5977.91 ± 4262.90	6692.91 ± 9003.95
	SDF-1a	61.91 ± 28.42	**111.60 ± 19.07 ***	91.84 ± 22.68	**126.60 ± 24.82 ***
	TNF-a	113.02 ± 24.42	**351.34 ± 73.37 ***	**322.20 ± 32.89 ***	**388.82 ± 11.70 ***
	TNF-b	104.96 ±36.48	135.34 ± 33.13	122.87 ± 26.41	105.74 ± 24.71
	TRAIL	41.68 ± 4.84	39.37 ± 10.53	38.95 ± 10.58	35.51 ± 8.81
	VEGF	299.25 ± 43.27	295.29 ± 34.71	246.06 ± 69.21	212.08 ± 45.15

	b-NGF	23.44 ± 1.99	24.37 ± 3.95	26.94 ± 4.69	28.49 ± 0.06
	bFGF	421.99 ± 31.45	440.64 ± 37.56	439.72 ± 57.48	438.48 ± 1.12
Interleukins	IL-1Ra	214.21 ± 54.52	**460.88 ± 45.29 ***	392.48 ± 76.10	**432.19 ± 99.44 ***
	IL-1α	98.89 ± 10.65	**135.18 ± 27.18 ***	**136.10 ± 18.39 ***	**243.96 ± 165.50 ***
	IL-1β	19.51 ± 1.86	21.07 ± 2.47	21.92 ± 2.23	20.58 ± 2.92
	IL-2	149.24 ± 4.19	150.22 ± 4.12	144.70 ± 8.08	151.69 ± 3.06
	IL-2Ra	119.07 ± 8.84	**171.21 ± 42.27 ***	**149.88 ± 2.97 ***	**164.16 ± 25.93 ***
	IL-3	13.24 ± 1.26	12.81 ± 1.33	13.42 ± 1.36	13.01 ± 0.49
	IL-4	12.36 ± 1.93	**16.66 ± 1.14 ***	**16.95 ± 1.34 ***	**19.26 ± 0.80 ***
	IL-5	395.84 ± 32.67	395.14 ± 28.46	376.96 ± 35.28	403.31 ± 12.56
	IL-6	31.00 ± 3.52	28.05 ± 5.85	30.48 ± 6.39	25.38 ± 1.37
	IL-7	164.45 ± 17.43	151.94 ± 7.01	151.83 ± 5.01	161.31 ± 15.47
	IL-8	35.75 ± 5.37	**24.24 ± 2.72 ***	**24.72 ± 1.71 ***	**23.63 ± 5.54 ***
	IL-9	21.04 ± 2.10	20.65 ± 3.02	18.35 ± 4.88	26.29 ± 5.97
	IL-10	11.22 ± 1.21	**15.84 ± 2.50 ***	13.64 ± 1.23	**18.39 ± 6.13 ***
	IL-12p40	626.63 ± 63.94	567.82 ± 82.43	551.69 ± 115.21	648.46 ± 15.70
	IL-12p70	274.80 ± 25.20	245.57 ± 57.42	281.88 ± 17.29	252.79 ± 59.12
	IL-13	24.16 ± 4.17	24.87 ± 6.43	23.50 ± 2.17	19.63 ± 1.79
	IL-15	1292.82 ± 12.72	1317.48 ± 41.93	1285.92 ± 14.96	1298.45 ± 27.51
	IL-16	99.38 ± 7.18	97.70 ± 9.64	101.96 ± 3.58	106.06 ± 2.07
	IL-17	195.56 ± 13.40	204.32 ± 12.40	217.41 ± 52.22	201.57 ± 10.87
	IL-18	69.20 ± 3.71	68.01 ± 4.73	70.35 ± 4.42	70.49 ± 1.17
Urine kidney damage markers	Calbindin	3.99 ± 4.44	**45.92 ± 38.44 ***	**26.21 ± 23.39 ***	**18.01 ± 14.16 ***
	GST-p	7.28 ± 8.61	6.59 ± 15.35	3.32 ± 4.82	5.70 ± 6.68
	IL-18	0.18 ± 0.37	0.05 ± 0.05	0.04 ± 0.06	0.08 ± 0.18
	KIM-1	3.45 ± 9.51	0.35 ± 0.35	0.53 ± 0.62	0.20 ± 0.21
	MCP-2	0.89 ± 1.20	1.16 ± 1.81	1.17 ± 1.85	0.35 ± 0.84
	Clusterin	3.69 ± 6.39	**17.11 ± 16.52 ***	**14.70 ± 12.30 ***	5.32 ± 4.84

* *p* adj. < 0.05 when compared with controls.
